# Reported practice patterns in the use of local antibiotics for fracture-related infection among SICOT respondents: an international survey

**DOI:** 10.1186/s12891-026-10585-7

**Published:** 2026-10-09

**Authors:** Gregor Bielesch, Conrad Ketzer, Ahmed Ellafi, Olivia Bohe, Peter Biberthaler, Philipp Rittstieg, Felix Karl-Ludwig Klingebiel, Roman Pfeifer, Georg Osterhoff, Stephan Frenzel, Sattar Alshryda, Arindam Banerjee, Stefan Huber-Wagner, Marc Hanschen, SICOT Global Research Network

**Affiliations:** 1 Department of Trauma Surgery, Klinikum rechts der Isar, TUM University Hospital, Technical University of Munich, Ismaninger Str. 22, 81675 Munich, Germany; 2 Department of Traumatology, University Hospital Zurich, University of Zurich, Zurich, Switzerland; 3 Department of Trauma and Orthopaedic Surgery, BG Klinikum Unfallkrankenhaus Berlin, Berlin, Germany; 4 Lehrstuhl für Traumatologie, Charité - Universitätsmedizin Berlin, CharitéCentrum für Orthopädie und Unfallchirurgie CC 9, Berlin, Germany; 5 Department of Orthopaedics and Trauma-Surgery, University Hospital Vienna, Medical University of Vienna, Vienna, Austria; 6 Pediatric Orthopaedics and Trauma, Al Jalila Children’s Speciality Hospital, Dubai, UAE; 7 NSH and NMH, Narayana Health Group of Hospitals, Kolkata, India; 8 Department of Trauma Surgery, Diakonie Klinikum Landkreis Schwäbisch Hall, Schwäbisch Hall, Germany

**Keywords:** Fracture-related infection, FRI, Local antibiotics, Infection management, Antibiotic carriers, SICOT

## Abstract

**Background:**

Local antibiotics are increasingly used in the management of fracture-related infection (FRI), but international practice patterns regarding their indications, antibiotic selection, and application methods remain heterogeneous and insufficiently standardised.

**Methods:**

An international survey was conducted among members of the Société Internationale de Chirurgie Orthopédique et de Traumatologie (SICOT). The web-based questionnaire included 16 items on indications, antibiotic selection, and application methods. Participation was voluntary and self-selected. Responses were analysed descriptively, with regional stratification performed for exploratory purposes. Of 118 started questionnaires, 87 were included in the analysis (73.7%); the overall response rate could not be calculated because the total number of invited SICOT members was unavailable.

**Results:**

87 surgeons from six continents participated, most with extensive clinical experience. The analysis revealed substantial heterogeneity in the frequency of local antibiotic use as well as in the selected antibiotic agents and application methods. An increase in local antibiotic use over the past decade was reported by 48/87 (55.2%) respondents. The main reported reasons for using local antibiotics were surgeon’s preference (44/87, 50.6%) and intraoperative findings (26/87, 29.9%).

**Conclusions:**

Among the surveyed SICOT respondents, local antibiotic use was reported across all represented regions, but substantial variation was observed in reported indications, antibiotic selection, and delivery methods. Given the unavailable invitation denominator and highly imbalanced regional sample sizes, these findings should be interpreted as exploratory and should not be considered evidence of treatment effectiveness or preferred practice.

**Supplementary information:**

The online version contains supplementary material available at https://doi.org/10.1186/s12891-026-10585-7.

## Background

Fracture-related infection (FRI) is one of the most serious complications following orthopaedic and trauma surgery. It leads to delayed bone healing, functional impairment, repeated revision procedures, and substantial socioeconomic burden [1, 2]. For specialised trauma centres, FRI represents a major clinical and organisational challenge.

To address these challenges, several international expert groups have developed consensus papers to standardise the definition and treatment principles of FRI. The 2018 international consensus definition established a diagnostic framework combining clinical, radiological, microbiological, and histopathological criteria [3]. Confirmatory criteria include isolation of the same pathogen from at least two deep tissue samples, a sinus tract or exposed implant, and purulent drainage, whereas suggestive criteria include clinical, laboratory, or radiological signs of infection. This definition has become the global reference standard for clinical diagnosis and research and was validated in 2022 [4].

Pesch et al. (2020) complemented this framework by introducing a conceptual model structuring FRI treatment into infection control, dead-space management, and reconstructive restoration [5]. Building on the 2018 consensus definition, the 2020 recommendations formulated a structured treatment concept for FRI [6]. Core principles include radical debridement, standardised tissue sampling, restoration of mechanical stability, meticulous dead-space management, and adequate soft-tissue coverage [7–9]. Systemic antibiotic therapy is recommended, while local antimicrobial therapy should be strongly considered.

Despite these recommendations, FRI management remains heterogeneous worldwide, with variability in indications, antibiotic selection, carrier materials, and duration of therapy. Local antibiotic strategies are increasingly described as promising adjuncts because they allow high drug concentrations at the infection site with limited systemic exposure while supporting eradication of biofilm and devitalised tissue [10–12]. McNally et al. introduced and validated a single-stage treatment paradigm for chronic osteomyelitis and FRI, demonstrating that radical debridement combined with local antibiotic carriers can achieve high infection control rates without routine staged procedures [13]. Consequently, local antibiotic therapy is increasingly considered an enabling factor for single-stage strategies in selected cases [14]. However, local antibiotic application may also promote selection of antibiotic-resistant strains, requiring careful agent selection and microbiological surveillance [15].

A broad range of local antibiotic delivery systems is available. Powder or solution-based applications, such as vancomycin powder, antibiotic irrigation, or local infusion, provide high-dose exposure at the surgical site [16–18]. Solid carriers including antibiotic-loaded pellets enable sustained antibiotic release and dead-space management [19]. Implant coatings and antibiotic-impregnated cancellous bone grafts combine antimicrobial activity with mechanical stability or biological reconstruction [20]. Non-resorbable materials such as polymethylmethacrylate (PMMA) beads remain widely used but require removal [21], whereas resorbable carriers including calcium sulphate or phosphate ceramics and collagen-based systems degrade over time and may support bone regeneration [22, 23]. This diversity reflects the clinical relevance of local antibiotic therapy but also the absence of standardised protocols.

Although reviews and consensus papers have addressed local antibiotic therapy in FRI, clear evidence-based recommendations regarding optimal indications, agents, and delivery systems remain lacking due to limited comparative clinical data [24]. Therefore, this study aims to describe international practice patterns in the use of local antibiotics for FRI and to identify areas of variation and uncertainty requiring further comparative clinical research.

## Methods

This study was designed as a survey aiming to assess current practices and attitudes regarding the use of local antibiotics in FRI cases. The questionnaire was developed on the basis of a review of current literature and clinical experience, reviewed by members of the Société Internationale de Chirurgie Orthopédique et de Traumatologie (SICOT) trauma committee for relevance, clarity, and content, and tested for technical functionality before distribution. The web-based survey was administered via the SoSci Survey platform (SoSci Survey GmbH, Munich, Germany) between April 14 and June 30, 2025. Active SICOT members were invited via the society’s email distribution list, and a reminder was sent after seven weeks. Voluntary participation was open to active SICOT members and was not restricted by hospital level, institutional FRI case volume, or predefined criteria of individual expertise. The survey was conducted as an open survey using a common link. Individual invitation links or participation codes were not used, and no specific mechanism for preventing duplicate submissions was implemented. IP addresses were not stored and cookies were not used. Respondents could review and modify their answers using the back function until final submission. No survey items were mandatory except for the data protection declaration, and no automated completeness checks were performed. Completion time was not used as an exclusion criterion.

A total of 87 questionnaires were submitted and included in the analysis. Four of the 87 submitted questionnaires contained unanswered items toward the end of the questionnaire. These questionnaires were retained, and all valid responses provided were included in the analyses. Accordingly, the denominator varied between individual questions according to the number of valid responses available for each item. As the exact number of SICOT members included in the mailing distribution list was unavailable, an overall response rate could not be calculated. Reporting of the web-based survey was aligned with the applicable items of the Checklist for Reporting Results of Internet E-Surveys (CHERRIES) [25].

The questionnaire comprised 16 items covering participant characteristics, frequency and indications of local antibiotic use in FRI, trends in utilisation, determinants of application, antibiotic selection, availability of infectious disease (ID) expertise and microbiological standards, and different methods of local antibiotic application (Supplementary material 1). Questionnaire items and response options were presented in a fixed order and were not randomized. All survey questions were presented to all participants irrespective of their reported frequency of local antibiotic use, and analyses were therefore based on the full cohort of 87 respondents where valid responses were available; otherwise, the number of valid respondents for the respective item is reported.

Free-text antibiotic entries were manually reviewed and standardised. Spelling variants were harmonised, brand names were converted to generic names where applicable, and individual agents were grouped by antimicrobial class. Multiple identifiable agents within a single entry were coded separately, while unclear or non-antibiotic entries were excluded.

Categorical variables are presented as absolute and relative frequencies. Continuous variables are summarised using pooled proportions, median, and range where appropriate. Data were analysed descriptively without formal hypothesis testing. Stratification by continent was performed for descriptive purposes only to illustrate regional trends. Analyses and visualisations were conducted using GraphPad Prism (GraphPad Software, San Diego, CA, USA).

## Results

### Participant demographics

The survey platform recorded 126 survey openings and 118 started questionnaires, of which 87 were completed and included in the analysis, corresponding to a completion rate of 73.7%. Most respondents were based in Asia (45/87, 51.7%), followed by Europe (21/87, 24.1%) and Africa (13/87, 14.9%), with fewer respondents from North America (4/87, 4.6%), Australia (3/87, 3.4%), and South America (1/87, 1.1%). The majority of respondents were male (81/87, 93.1%).

Most participants were senior surgeons: 60/87 (68.9%) reported more than 10 years of experience and 10/87 (11.5%) less than five years. Consultants/attendings accounted for 54/87 (62.1%) of respondents, followed by heads of department 23/87 (26.4%), fellows 9/87 (10.3%), and residents 1/87 (1.1%).

Regarding institutional background, 38/87 (43.7%) worked at Level I trauma centres or university hospitals, 21/87 (24.1%) at Level II centres, and 21/87 (24.1%) at Level III or general hospitals, with few respondents from Level IV 5/87 (5.7%) or Level V 2/87 (2.3%) facilities. Detailed demographics are provided in Table 1.

**Table 1:** Participant demographics

Demographics	Africa	Asia	Australia	Europe	North America	South America	Inter-national
n	13	45	3	21	4	1	87
Male gender, n (%)	13 (100)	44 (97.8)	3 (100)	19 (90.5)	2 (50)	0	81 (93.1)
Professional position, n (%)							
Resident	0	1 (2.2)	0	0	0	0	1 (1.1)
Fellow	4 (30.8)	4 (8.9)	0	1 (4.8)	0	0	9 (10.3)
Consultant / Attending	5 (38.5)	31 (68.9)	3 (100)	12 (57.1)	3 (75)	0	54 (62.1)
Head of Department	4 (30.8)	9 (20)	0	8 (38.1)	1 (25)	1 (100)	23 (26.4)
Professional experience, n (%)							
< 5 years	2 (15.4)	8 (17.8)	0	0	0	0	10 (11.5)
5–10 years	4 (30.8)	10 (22.2)	0	2 (9.5)	1 (25)	0	17 (19.5)
> 10 years	7 (53.8)	27 (60)	3 (100)	19 (90.5)	3 (75)	1 (100)	60 (69)
Type of hospital, n (%)							
Level I Trauma Centre/ University hospital	7 (53.8)	21 (46.7)	0	9 (42.9)	0	1 (100)	38 (43.7)
Level II Trauma Centre	2 (15.4)	8 (17.8)	2 (66.7)	8 (38.1)	1 (25)	0	21 (24.1)
Level III Trauma Centre/ General care hospital	3 (23.1)	13 (28.9)	1 (33.3)	2 (9.5)	2 (50)	0	21 (24.1)
Level IV Trauma Centre	1 (7.7)	2 (4.4)	0	1 (4.8)	1 (25)	0	5 (5.7)
Level V Trauma Centre/ Primary care hospital	0	1 (2.2)	0	1 (4.8)	0	0	2 (2.3)

### Local antibiotic use in FRI

**Fig. 1:**
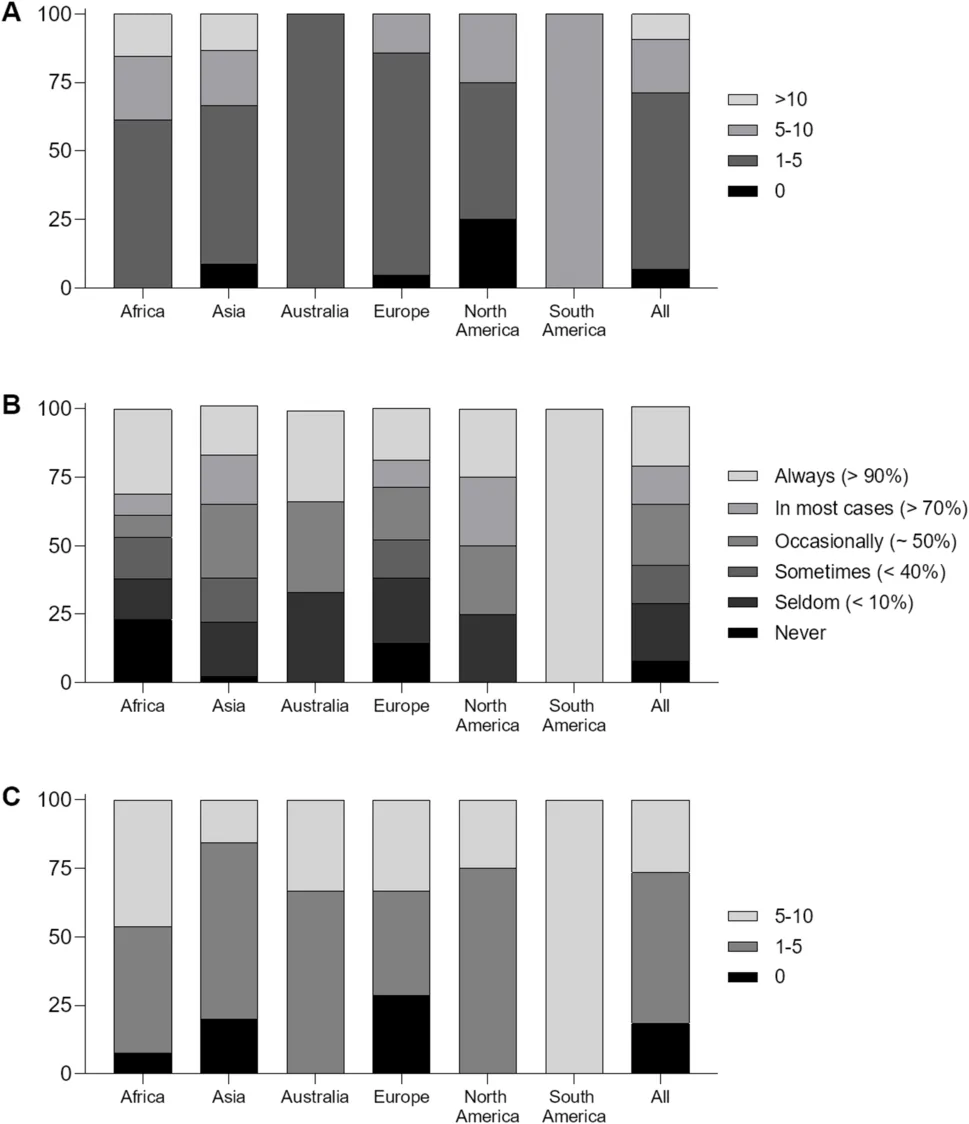
International patterns of local antibiotic use in fracture-related infection (FRI). **A** Number of FRIs treated per centre per month. **B** Frequency of local antibiotic use in FRI treatment. **C** Reported change in the use of local antibiotics for FRI treatment over the past decade. Categorical response distributions are shown as relative frequencies and are stratified by continent. Numbers of respondents were: Africa (*n* = 13), Asia (*n* = 45), Australia (*n* = 3), Europe (*n* = 21), North America (*n* = 4), and South America (*n* = 1)

Across regions, the most frequently reported number of treated FRIs was 1–5 cases per month by (56/87, 64.4%; Fig. 1A). Higher case volumes (> 10 cases per month) were reported mainly by centres in Asia and Africa.

Use of local antibiotics varied substantially among centres (Fig. 1B), demonstrating the absence of a uniform practice pattern across and within regions. While 19/87 (21.8%) respondents reported always using local antibiotics, 25/87 (28.7%) reported seldom or never using them. The median category of reported use was ‘occasionally’.

More than half of respondents (48/87, 55.2%) reported increased use of local antibiotics during the past decade (Fig. 1C), whereas most others indicated no change and 16/87 (18.3%) reported decreased use.

### Determinants of local antibiotic application

**Fig. 2:**
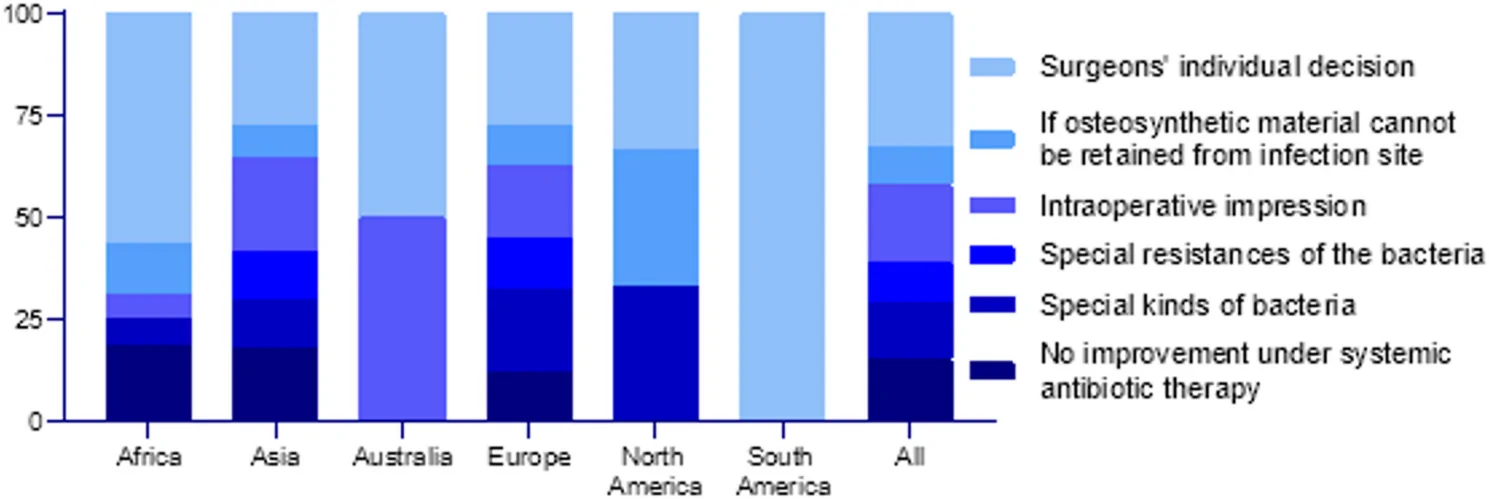
Reported key factors influencing the decision to use local antibiotics in FRI treatment. Stratification by continent. Percentages represent the relative frequency of each indication among all indications selected within a region. Multiple responses were allowed; therefore, values reflect the distribution of reported indications rather than the proportion of respondents selecting a given indication. Numbers of respondents were: Africa (*n* = 13), Asia (*n* = 45), Australia (*n* = 3), Europe (*n* = 21), North America (*n* = 4), and South America (*n* = 1)

The most frequently reported indications for local antibiotic use were the surgeon’s individual decision (chosen by 44 of 87 respondents, 50.6%), intraoperative findings (26/87, 29.9%), and insufficient response to systemic antibiotics (21/87, 24.1%; Fig. 2). Less frequently cited reasons included specific pathogens (19/87, 21.8%), antimicrobial resistance (13/87, 14.9%), and the inability to remove osteosynthetic material from the infection site (13/87, 14.9%). Multiple responses were permitted, and percentages therefore do not sum to 100%.

### Availability of ID expertise and microbiological standards

Access to ID specialists was generally high (78/87, 89.7%; Table 2). In Europe all respondents reported access. Although access was also common in Asia (37/45, 82.2%) and Africa (12/13, 92.3%), consultation rates were lower (21/37, 56.8% and 3/12, 25.0%). Overall, 52/78 (66.7%) centres with access to ID specialists reported a consultation standard.

**Table 2:** Availability and consultation of infectious diseases (ID) specialists and preparation of standardised antibiotic susceptibility profiles in FRI treatment, stratified by continent

	Africa	Asia	Australia	Europe	North America	South America	Inter-national
**ID specialists, ** ***n*** ** (%)**							
Availability	12/13 (92.3)	37/45 (82.2)	3/3 (100)	21/21 (100)	4/4 (100)	1/1 (100)	78/87 (89.7)
Consultation (where available)	3/12 (25)	21/37 (56.8)	3/3 (100)	20/21 (95.2)	4/4 (100)	1/1 (100)	52/78 (66.7)
**Antibiotic susceptibility profile preparation standard (%)**							
	7/13 (53.8)	31/45 (68.9)	2/3 (66.7)	17/21 (81)	3/4 (75)	1/1 (100)	61/87 (70.1)

ID specialist availability was derived from the response to the question whether the department consults ID specialists: “Yes” and “No” indicated that consultation was possible, whereas “Not possible” indicated that consultation was unavailable. ID specialist consultation was assessed among respondents for whom consultation was possible. Values are presented as n/N (%)

Standardised antibiotic susceptibility testing protocols were reported by 61/87 (70.1%) of centres internationally. Rates ranged from 7/13 (53.8%) in Africa to 17/21 (81%) in Europe.

### Empiric antibiotic spectrum and locally applied antibiotics

**Fig. 3:**
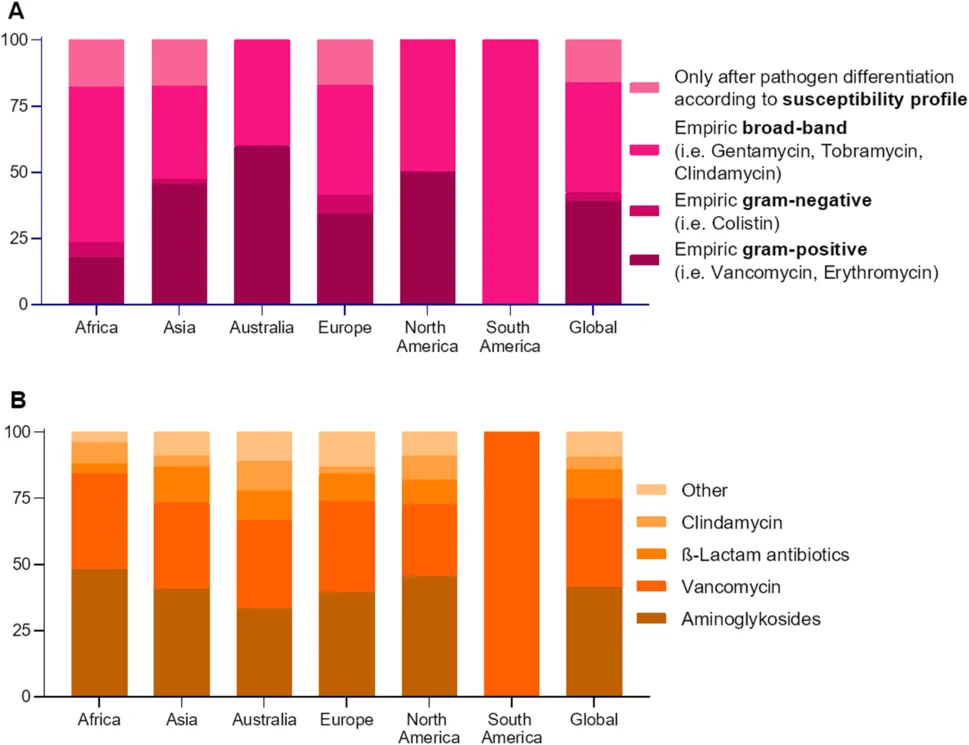
Antibiotic treatment strategies in fracture-related infection (FRI). **A** Empiric antibiotic spectrum used as first-line treatment, stratified by continent, showing that broad-spectrum regimens and gram-positive coverage are the most commonly reported initial approaches. **B** Most frequently used local antibiotic groups in FRI treatment, stratified by continent, highlighting aminoglycosides and vancomycin as the predominant agents internationally. Percentages represent the proportion of all selected agents; multiple selections were allowed. Numbers of respondents were: Africa (*n* = 13), Asia (*n* = 45), Australia (*n* = 3), Europe (*n* = 21), North America (*n* = 4), and South America (*n* = 1)

Initial antibiotic treatment strategies were broadly similar across regions (Fig. 3A). 113 selections were given by 87 respondents. Broad-spectrum regimens (47/113, 41.6%) and empiric gram-positive coverage (44/113, 38.9%) were most frequently selected. Only 18/113 (15.9%) reported initiating treatment after pathogen identification, whereas empiric gram-negative coverage alone was rare (4/113, 3.5%).

Vancomycin and aminoglycosides were the most frequently used local antibiotics internationally (Fig. 3B; Table 3). Among aminoglycosides, gentamicin was the predominant agent (59 mentions, 32.4% of 182 responses), followed by tobramycin (11/182, 6%), while vancomycin was mentioned in 61/182 responses (33.5%).

**Table 3:** Locally applied antibiotic agents used in the treatment of fracture-related infection (FRI)

Most commonly used local antibiotics		Mentions (*n*)	Proportion mentioned (%)
**Aminoglycosides**		**75**	**41.1**
	Gentamicin	59	32.4
	Tobramycin	11	6.0
	Amikacin	3	1.6
	Streptomycin	2	1.1
**Glycopeptides**		**61**	**33.5**
	Vancomycin	61	33.5
**β-Lactam antibiotics**		**20**	**11.0**
	Cephalosporins	14	7.7
	Penicillins	6	3.3
**Lincosamides**		**9**	**4.9**
	Clindamycin	9	4.9
**Rifamycins**		**5**	**2.7**
	Rifampicin	5	2.7
**Polymyxins**		**4**	**2.2**
	Colistin	4	2.2
**Fluoroquinolones**		**4**	**2.2**
	Ciprofloxacin	4	2.2
**Oxazolidinones**		**1**	**0.5**
	Linezolid	1	0.5
**Carbapenems**		**1**	**0.5**
	Meropenem	1	0.5
**Nitroimidazoles**		**1**	**0.5**
	Metronidazole	1	0.5
**Glycylcyclines**		**1**	**0.5**
	Tigecycline	1	0.5

A total of 186 antibiotic mentions were obtained from 83 respondents. Respondents could report up to three antibiotics. 4 entries were excluded because they were unclear, non-specific, or not antibiotics. Percentages represent the proportion of the 182 valid antibiotic mentions; respondents could report up to three antibiotics

Other antibiotics were used less frequently, including β-lactams (20/182, 11%), mainly cephalosporins (14/182, 7.7%) and penicillins (6/182, 3.3%). Additional agents included clindamycin (9/182, 4.9%), rifampicin (5/182, 2.7%), colistin (4/182, 2.2%), and ciprofloxacin (4/182, 2.2%), while linezolid, meropenem, metronidazole, and tigecycline were each mentioned once (1/182, 0.5%).

### Integration of local antibiotics into systemic therapy and treatment strategy

**Fig. 4:**
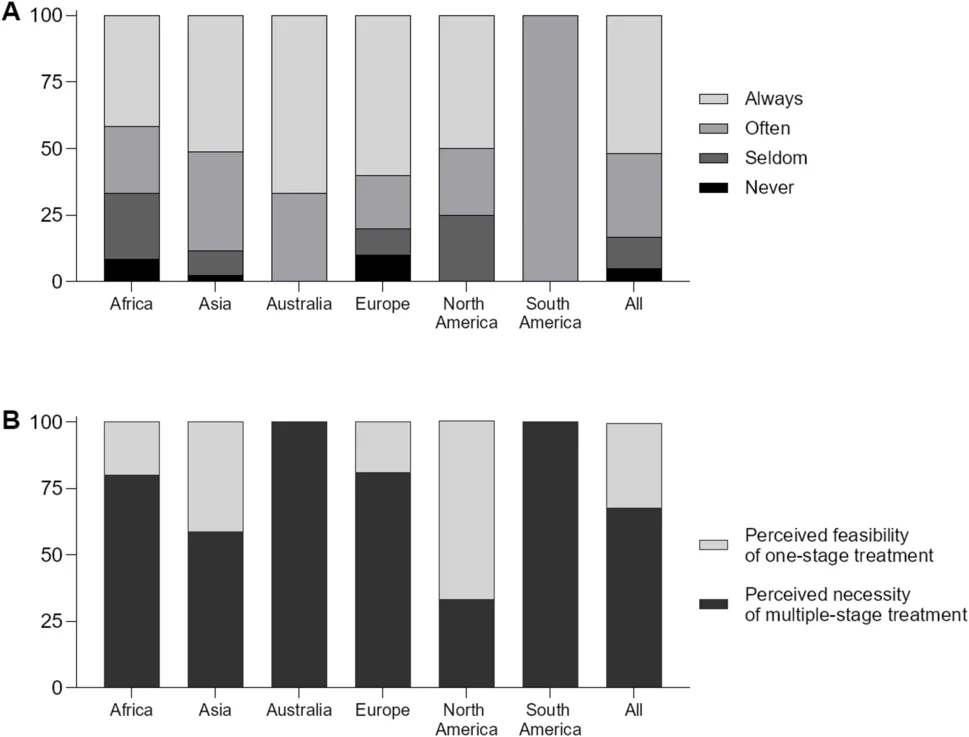
Role of local antibiotics in systemic therapy and treatment strategy in fracture-related infection (FRI). **A** Frequency of combining local and systemic antibiotics. **B** Respondents’ assessment of the role of local antibiotics in one-stage versus multiple-stage treatment. The response option “I do not use local antibiotics” was excluded from the graphical presentation. Categorical response distributions are shown as relative frequencies and are stratified by continent. Respondents by continent: Africa (*n* = 13), Asia (*n* = 45), Australia (*n* = 3), Europe (*n* = 21), North America (*n* = 4), and South America (*n* = 1)

Among the 83 respondents who provided valid responses to this section, combination therapy with systemic antibiotics was common (Fig. 4A). Internationally, 43/83 (51.8%) of respondents reported always and 26/83 (31.3%) often combining systemic and local antibiotics, whereas only a minority reported seldom (10/83, 12%) or never (4/83, 4.8%) using both approaches. 4 participants did not answer this item.

Among respondents using local antibiotics in the treatment of FRI, 48/71 (67.6%) indicated that the use of local antibiotics does not eliminate the need for multiple-stage treatment, whereas 23/71 (32.4%) reported that the use of local antibiotics had changed their practice and allowed for one-stage treatment options (Fig. 4B).

### Application types of local antibiotics

**Fig. 5:**
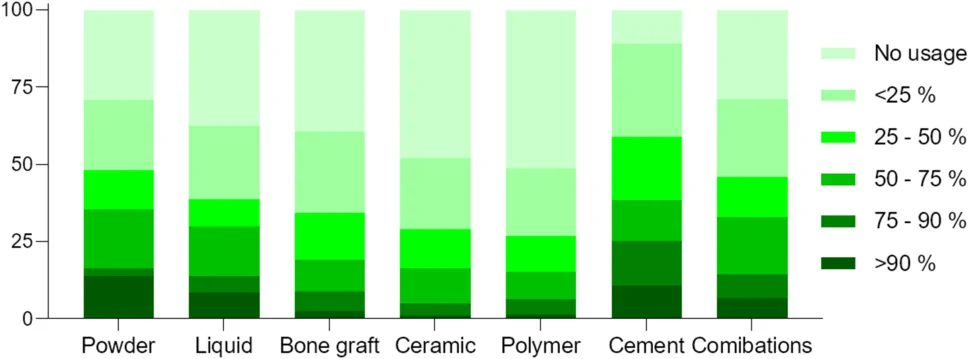
Application types of local antibiotics used for FRI. 100% stacked bar charts showing the distribution of responses for each application method of all 87 respondents. Segments represent the proportion of respondents selecting the respective usage category (no usage, < 25%, 25–50%, 50–75%, 75–90%, > 90% of cases). Application methods were grouped as follows: Powder (powder application of antibiotics into the wound), Liquid (liquid application of antibiotics into the wound), Bone graft (antibiotic-impregnated autologous or allogenic bone graft), Ceramic (synthetic resorbable ceramic bone replacement materials with antibiotic elution), Polymer (synthetic resorbable polymer bone replacement materials with antibiotic elution), Cement (non-resorbable materials/cement, e.g. polymethylmethacrylate (PMMA), with antibiotic elution), and Combinations (combinations of the above application methods)

Figure 5 presents the distribution of reported usage frequencies for each local antibiotic application method. Considerable variation was observed across all application types. Non-resorbable materials/cement carriers, powder and liquid application were among the most frequently reported approaches, whereas polymer-based carriers and ceramic carriers were used less consistently. No single application method predominated across all respondents.

## Discussion

FRI remains one of the most challenging complications in orthopaedic trauma surgery and continues to impose substantial clinical and socioeconomic burden worldwide [2]. The present international survey provides insight into how local antibiotic therapy is currently integrated into routine FRI management and highlights considerable variation in clinical practice.

Overall, the findings suggest substantial heterogeneity in the use of local antibiotics for FRI despite the availability of international consensus definitions and treatment principles [3, 6]. Across all surveyed regions, the reported frequency of use ranged widely, from centres that rarely apply local antibiotics to those that report routine use. At the same time, approximately half of respondents indicated an increased utilisation during the past decade, suggesting growing international interest in local antibiotic therapy as an adjunct in infection management. Even though the reported practices provide no information on effectiveness or clinical superiority, they indicate that local antibiotic treatment is widely used in FRI management, while its implementation remains far from standardised.

One of the most striking observations of the survey is the degree of variability in clinical decision-making. A substantial proportion of respondents reported that the decision to apply local antibiotics is primarily based on the surgeon’s individual judgement or intraoperative findings. This finding may reflect the limited availability of robust comparative evidence regarding optimal indications, substances, and delivery systems. Although international consensus recommendations emphasise the importance of local antibiotic therapy as part of a comprehensive treatment strategy, including radical debridement, fracture stabilisation, dead-space management, and systemic antibiotic therapy, they intentionally avoid prescribing specific indications or carrier systems [6, 24]. In this context, the variability observed in the present survey appears to reflect the flexibility of existing recommendations rather than non-adherence to them. Nevertheless, the results underline that clinical practice is currently guided largely by individual experience and local institutional structures.

The survey showed considerable heterogeneity in initial antibiotic selection, with individual respondents reporting a wide variety of antibiotic regimens before definitive microbiological results became available. Current treatment concepts recommend early empiric therapy targeting the most probable pathogens, followed by adjustment according to microbiological findings [1, 2]. Vancomycin and aminoglycosides, particularly gentamicin, were the most frequently reported local antibiotics, reflecting their antimicrobial spectrum, elution characteristics, and compatibility with commonly used carriers [11, 16]. Notably, evidence for carrier-free (“naked”) antibiotics such as vancomycin powder in established FRI remains limited, as available clinical studies predominantly address prophylactic use during fracture fixation [16, 18]. The additional substances listed in Table 3 reflect reported practice and should not be interpreted as recommended options. In particular, inappropriate use of rifampicin may promote rapid emergence of resistance, while broad-spectrum reserve antibiotics such as meropenem may exert unnecessary selection pressure [15].

Another important observation concerns the integration of multidisciplinary expertise into infection management. Although access to ID specialists was reported by most respondents, consultation rates varied considerably between regions, indicating that availability alone does not ensure routine integration into FRI care. Structured multidisciplinary pathways may improve interpretation of microbiological findings, antimicrobial selection, coordination of local and systemic treatment, and consistency of clinical decision-making. International recommendations emphasise multidisciplinary collaboration to optimise antibiotic stewardship and resistance control [15, 26]. Local delivery may result in declining or unpredictable antibiotic concentrations, particularly during prolonged elution or persistent wound leakage, potentially exposing residual bacteria to subinhibitory levels and favouring resistant or persistent phenotypes, including small-colony variants. Local antibiotics should therefore be selected according to microbiological findings whenever possible and regarded as an adjunct rather than a substitute for adequate surgical and systemic treatment. Differences in access to ID expertise and standardised microbiological diagnostics may influence the implementation of these principles across health-care systems [2, 27].

The survey findings also provide perspective on the ongoing discussion regarding one-stage versus multi-stage treatment concepts in FRI. While most respondents indicated that a staged approach remains necessary in their clinical practice, a considerable minority reported that local antibiotic therapy may facilitate single-stage procedures in selected cases. This observation aligns with an increasing number of reports describing favourable outcomes with single-stage treatment strategies that combine radical debridement with high local antibiotic concentrations and optimised soft-tissue management [10, 12, 14, 17, 21, 28]. However, these approaches typically require careful patient selection, reliable microbiological diagnostics, and the availability of suitable carrier systems, conditions that may not be present in all treatment environments. The comparatively limited proportion of centres applying such strategies in this survey therefore likely reflects the current stage of clinical adoption rather than a lack of interest in the concept.

Beyond these therapeutic considerations, the survey highlights broader structural differences between health-care systems. Variability in consultation practices, microbiological infrastructure, and availability of carrier materials suggests that local treatment strategies are influenced not only by clinical reasoning but also by institutional resources and organisational frameworks. These disparities may be particularly relevant in low- and middle-income regions, where access to specialised expertise or advanced biomaterials may be limited [27, 29]. Such differences emphasise the importance of considering health-system context when interpreting international practice patterns.

Several limitations must be considered. Participation was limited to the SICOT network and was not restricted by predefined criteria of individual expertise, institutional case volume, or hospital level. Accordingly, the sample represents a heterogeneous group of surgeons rather than a formal expert consensus, which may limit generalisability. The absence of an exact invitation denominator prevented calculation of an overall response rate and limits assessment of potential non-response bias. Because a common link was used without mechanisms to prevent or identify duplicate submissions, duplicate participation cannot be excluded.

Furthermore, the comparatively small number of respondents from North and South America and Australia, as well as the high proportion of respondents from India among those from Asia, consistent with lower SICOT membership in these areas, limits the reliability of regional comparisons, which should be interpreted as exploratory only. As with any questionnaire-based study, the responses represent self-reported estimates and individual perceptions rather than objective institutional data. In addition, predefined response categories may have constrained the range of possible answers and simplified complex clinical decision-making processes. Finally, the descriptive cross-sectional design does not allow conclusions regarding causal relationships or treatment outcomes. Consequently, the findings should be interpreted as exploratory observations describing current practice patterns rather than definitive evidence regarding optimal treatment strategies.

## Conclusion

This international survey illustrates that local antibiotic therapy is widely used in FRI management, while considerable heterogeneity exists regarding indications, antibiotic selection, carrier systems, and integration into overall treatment concepts. These findings describe current practice and identify important evidence gaps but do not establish preferred treatment strategies. Prospective comparative studies are needed to provide the evidence required for future recommendations and greater standardisation of clinical practice.

## Supplementary information

Supplementary Material 1

## Data Availability

Data, additional materials and code are available from the corresponding author upon reasonable request.

## Abbreviations

FRI: Fracture-related infection
ID: Infectious Disease
SICOT: Société Internationale de Chirurgie Orthopédique et de Traumatologie
