# Supplementary material for: Reported practice patterns in the use of local antibiotics for fracture-related infection among SICOT respondents: an international survey
Source: BMC Musculoskelet Disord. 2026 Oct 9;27:870. doi: 10.1186/s12891-026-10585-7 (PMC13670668; doi:10.1186/s12891-026-10585-7)
Supplement: Supplementary file 1 [file 12891_2026_10585_MOESM1_ESM.docx]

**Supplementary Material 1: Survey questionnaire**

Data protection consent [mandatory consent required to proceed]

a. I agree to the data protection policy.

1. Gender [single choice]
   a. Male
   b. Female
   c. Other
2. Level of education/training [single choice]
   a. Intern
   b. Resident
   c. Fellow
   d. Consultant/Attending
   e. Head of Department
3. Professional experience (in years) [single choice]
   a. < 5 years
   b. 5–10 years
   c. > 10 years
4. Type of hospital [single choice]
   a. Level I Trauma Centre / University hospital
   b. Level II Trauma Centre
   c. Level III Trauma Centre / General care hospital
   d. Level IV Trauma Centre
   e. Level V Trauma Centre / Primary care hospital
5. Country of your current employment [free-text field]
6. Number of fracture-related infection cases treated per month [free-text field]
7. In case of fracture-related infection, how often are local antibiotics used? [single choice]
   a. Always (> 90 % of cases)
   b. In most cases (> 60 % of cases)
   c. Occasionally (~ 50 % of cases)
   d. Sometimes (< 40 % of cases)
   e. Seldom (< 10 % of cases)
   f. Never
8. In your institution, how do you rate the trend of local antibiotic usage in cases with fracture-related infection within the last 10 years [single choice]
   a. Increase
   b. No change
   c. Decrease
9. In case of fracture-related infection, which findings are key factors that lead to adding local antibiotics to the treatment strategy? [multiple choice]
   a. No improvement under systemic antibiotic therapy
   b. Special kinds of bacteria
   c. Special resistances of the bacteria
   d. Intraoperative impression
   e. If osteosynthetic material cannot be retained from the infection site
   f. Surgeon’s individual decision
   g. Other: [free-text field]
10. In case of fracture-related infection, does your department consult infectiology specialists (i.e., the department of infectiology) to create an individual treatment plan? [single choice]
    a. Yes
    b. No
    c. Not possible
11. In case of usage of local antibiotics in fracture-related infection: Please specify the first-line (pre-antibiogram) local antibiotic treatment. [multiple choice]
    a. Calculated gram-positive (i.e. Vancomycin, Erythromycin)
    b. Calculated gram-negative (i.e. Colistin)
    c. Calculated broad-band (i.e. Gentamicin, Tobramycin, Clindamycin)
    d. Only after pathogen differentiation according to antibiogram
12. In case of usage of local antibiotics in fracture-related infection: Is an antibiogram / resistogram prepared as part of the standard procedure, and is the local antibiotics treatment adjusted accordingly? [single choice]
    a. Yes
    b. No
13. In the case of usage of local antibiotics: Please list the most commonly used antibiotics (up to 3) in your institution [free-text field, up to three entries]
14. In case of usage of local antibiotics in fracture-related infection: How often are the following application types of local antibiotics used in your institution? [one response per application type: no usage; < 25 %; 25 – 50 %; 50 – 75 %; 75 – 90 %; > 90 %]
    a. **Powder application** of antibiotics into the wound
    b. **Liquid application** of antibiotics into the wound
    c. Antibiotic-impregnated **autologous or allogenic bone graft**
    d. Synthetic resorbable **ceramic bone replacement** materials with antibiotic elution
    e. Synthetic resorbable **polymer** **bone replacement** materials with antibiotic elution
    f. **Non-resorbable materials/ cement** (i.e. PMMA) with antibiotic elution
    g. **Combinations** of the above
15. How often are local antibiotics combined with systemic antibiotic administration in the treatment of fracture-related infection? [single choice]
    a. Always
    b. Often
    c. Seldom
    d. Never
16. Kindly assess the usage of local antibiotics in fracture-related infection in relation to one-stage versus multiple-stage surgical approaches. [single choice]
    a. The usage of local antibiotics in fracture-related infection changed my practice and allowed for one-stage treatment options.
    b. The usage of local antibiotics in fracture-related infection does not eliminate the need for multiple-stage treatment.
    c. I do not use local antibiotics in the treatment of fracture-related infections.

**Supplementary Material 2: List of countries of participants**

| **Country of current employment** | **Participants (n)** | **Participants (%)** |
| --- | --- | --- |
| Afghanistan | 1 | 1.1 % |
| Aruba | 1 | 1.1 % |
| Australia | 3 | 3.4 % |
| Austria | 1 | 1.1 % |
| Azerbaijan | 1 | 1.1 % |
| Bangladesh | 1 | 1.1 % |
| Belgium | 2 | 2.3 % |
| Brazil | 1 | 1.1 % |
| British Indian Ocean Territory | 1 | 1.1 % |
| China | 2 | 2.3 % |
| Egypt | 1 | 1.1 % |
| Ethiopia | 1 | 1.1 % |
| France | 2 | 2.3 % |
| Germany | 1 | 1.1 % |
| Ghana | 3 | 3.4 % |
| Greece | 3 | 3.4 % |
| Hungary | 1 | 1.1 % |
| India | 19 | 21.8 % |
| Indonesia | 1 | 1.1 % |
| Iraq | 2 | 2.3 % |
| Italy | 1 | 1.1 % |
| Jordan | 1 | 1.1 % |
| Kenya | 1 | 1.1 % |
| Libya | 1 | 1.1 % |
| Malaysia | 1 | 1.1 % |
| Mexico | 3 | 3.4 % |
| Nepal | 5 | 5.7 % |
| Nigeria | 4 | 4.6 % |
| Pakistan | 4 | 4.6 % |
| Romania | 3 | 3.4 % |
| Rwanda | 1 | 1.1 % |
| Saudi Arabia | 2 | 2.3 % |
| Serbia | 1 | 1.1 % |
| Singapore | 1 | 1.1 % |
| South Africa | 1 | 1.1 % |
| South Korea | 1 | 1.1 % |
| Switzerland | 3 | 3.4 % |
| United Kingdom | 3 | 3.4 % |
| Vietnam | 1 | 1.1 % |
| Yemen | 1 | 1.1 % |
